# EGFP insertional mutagenesis reveals multiple FXR2P fibrillar states with differing ribosome association in neurons

**DOI:** 10.1242/bio.046383

**Published:** 2019-08-15

**Authors:** Emily E. Stackpole, Michael R. Akins, Maria Ivshina, Anastasia C. Murthy, Nicolas L. Fawzi, Justin R. Fallon

**Affiliations:** 1 Department of Neuroscience, Brown University, Providence, RI 02912, USA; 2 Department of Biology, Drexel University, Philadelphia, PA 19104, USA; 3 Program in Molecular Medicine, University of Massachusetts Medical School, Worcester, MA 01605, USA; 4 Graduate Program in Molecular Biology, Cell Biology, and Biochemistry, Brown University, Providence, RI 02912, USA; 5 Department of Molecular Pharmacology, Physiology, and Biotechnology, Brown University, Providence, RI 02912, USA

**Keywords:** RNA-binding protein, Fragile X syndrome, RNA granule, Local protein synthesis, Low complexity

## Abstract

RNA-binding proteins (RBPs) function in higher-order assemblages such as RNA granules to regulate RNA localization and translation. The Fragile X homolog FXR2P is an RBP essential for formation of neuronal Fragile X granules that associate with axonal mRNA and ribosomes in the intact brain. However, the FXR2P domains important for assemblage formation in a cellular system are unknown. Here we used an EGFP insertional mutagenesis approach to probe for FXR2P intrinsic features that influence its structural states. We tested 18 different in-frame FXR2P^EGFP^ fusions in neurons and found that the majority did not impact assemblage formation. However, EGFP insertion within a 23 amino acid region of the low complexity (LC) domain induced FXR2P^EGFP^ assembly into two distinct fibril states that were observed in isolation or in highly-ordered bundles. FXR2P^EGFP^ fibrils exhibited different developmental timelines, ultrastructures and ribosome associations. Formation of both fibril types was dependent on an intact RNA-binding domain. These results suggest that restricted regions of the LC domain, together with the RNA-binding domain, may be important for FXR2P structural state organization in neurons.

## Abstract (precis)

**Summary:** A mutagenesis study reveals that the higher-order structural states of the RBP FXR2P in neurons can be regulated by manipulation of the LC and RNA-binding domains.

## INTRODUCTION

Neurons are highly elaborate cells that mount specific and dynamic responses to a range of stimuli within their vast subdomains. One important mechanism for such spatiotemporal control is local protein synthesis, where mRNAs are targeted to specific domains and can be rapidly translated in response to nearby cues. This process has long been appreciated in dendrites (Holt and Schuman, 2013; Sutton and Schuman, 2006) and recent evidence indicates that analogous translational machinery is also present in axons (Zheng et al., 2001; Christie et al., 2009; Taylor et al., 2009; Korsak et al., 2016; Shigeoka et al., 2016; Akins et al., 2017; Batista et al., 2017). Local protein synthesis is in part controlled by RNA-binding proteins (RBPs) that can influence transcript fate through regulation of RNA biogenesis, localization, translation and degradation (Kapeli and Yeo, 2012; Darnell, 2013; Calabretta and Richard, 2015). Further, RBP alteration or loss is the basis for a wide range of neurological diseases including Fragile X syndrome, frontotemporal dementia, spinal muscular atrophy, myotonic dystrophy and amyotrophic lateral sclerosis (Liu-Yesucevitz et al., 2011; Wang et al., 2016).

RBPs are integral components of RNA granules, a class of cytosolic ‘assemblages’ that are critical regulators of mRNA transport, targeting and local translation (Kiebler and Bassell, 2006; Toretsky and Wright, 2014; Buchan, 2014; Calabretta and Richard, 2015; Nielsen et al., 2016). Formation of RNA granules is dependent upon the low complexity (LC) domains that are present in a majority of RBPs (Kato et al., 2012; Han et al., 2012; Weber and Brangwynne, 2012; King et al., 2012; Guo and Shorter, 2015). LC domains are intrinsically disordered, but are dynamic and can promote self-association to organize proteins into highly concentrated states, possibly via changes in LC conformation (Tompa, 2012; Boke et al., 2016; Boeynaems et al., 2018)*.* Therefore, some RBPs harboring LC domains are capable of existing in multiple structural conformations – with an ordered configuration presumably underlying a unique functional state. For example, Xvelo can assemble into an amyloid-like state in the Balbiani body in *Xenopus* (Boke et al., 2016).

RBP-RNA associations are a driving force behind LC domain fibrillization and complex macromolecular organization (Weber and Brangwynne, 2012; Schwartz et al., 2013; Burke et al., 2015; Molliex et al., 2015; Elbaum-Garfinkle et al., 2015). The importance of the strict regulation of the structural states of RBPs is highlighted by the large number of LC domain mutations that cause neurodegenerative diseases (Kim et al., 2013; Maziuk et al., 2017; Harrison and Shorter, 2017; Mackenzie et al., 2017). Therefore, characterizing the role of LC domains in defining RBP structural states and assemblage formation is an important step towards elucidating the mechanisms that control proper local translation as well as RBP-mediated pathogenesis in the nervous system.

FXR2P (Fragile X related protein 2) is a member of the Fragile X-related family of RNA-binding proteins that also includes FXR1P and FMRP (Fragile X mental retardation protein). Variants in all three contribute to autism risk, with loss of FMRP causing the autism-related disorder Fragile X syndrome (Schluth-Bolard et al., 2010; Stepniak et al., 2015). All three FXR proteins have equivalent RNA-binding properties via the KH domains (Darnell et al., 2009). However, FXR2P is unique in the Fragile X family in being the essential component of Fragile X granules (FXGs), a class of endogenous RNA granules present within axonal arbors of a subset of stereotyped neurons (Akins et al., 2012, 2017). FXGs can associate with ribosomes, FMRP and mRNAs encoding proteins important for neuronal plasticity (Christie et al., 2009; Akins et al., 2017; Chyung et al., 2018).

These observations suggest that FXR2P harbors intrinsic features that contribute to its function of promoting higher-order assemblage formation in neurons. The amino terminal region of FXR2P is >90% identical to FMRP and contains two KH and Tudor RNA-binding domains as well as nuclear import and export domains (Zhang et al., 1995; Darnell et al., 2009; Adams-Cioaba et al., 2010). However, FXR2P diverges from the rest of the Fragile X family in key respects. First, the FXR2P carboxyl-terminal domain has been reported to contain nucleolar-targeting signals (Tamanini et al., 1999, 2000). Second, we previously showed that FXR2P is the sole family member that is N-myristoylated, a modification that regulates its axonal distribution but not granule assembly (Stackpole et al., 2014). An intriguing possibility for FXR2P regulation of assemblage formation is the carboxy-terminal LC domain (Kato et al., 2012). Notably, while this domain is present within all three Fragile X proteins, the FXR2P LC domain is divergent and shares only ∼19 and ∼37% sequence identity with the comparable regions of FMRP and FXR1P.

Here we sought to define the features of FXR2P that influence its ability to assemble into higher-order structural states in cells. We used an insertional EGFP strategy that revealed discrete sites within the LC domain where EGFP insertion transitions FXR2P^EGFP^ into multiple unique fibrillar states when expressed in neurons. FXR2P^EGFP^ assembles into both isolated and bundled fibrillar states that exhibit different developmental timelines and ultrastructures. Notably, mRNA and ribosome association vary between FXR2P^EGFP^ fibril types and a functional KH2 RNA-binding domain is required for fibril formation. Further, we find that deletion of the LC domain results in a loss of FXR2P assemblages. These results, in combination with bioinformatic predictors, suggest that the RNA-binding domains and discrete regions of the LC domain regulate the structural and compositional states of FXR2P in neurons.

## RESULTS

### An insertional mutagenesis screen to identify FXR2P domains important for assembly into higher-order structural states

**Fig. 1.**
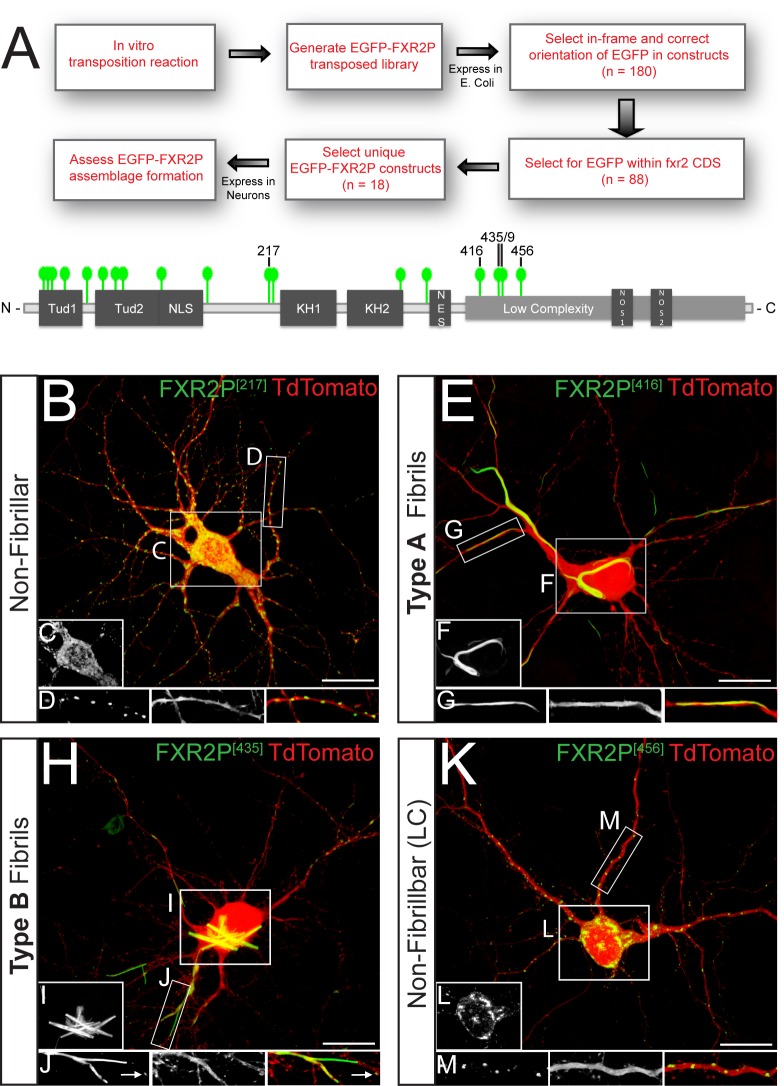
**Unbiased insertional mutagenesis screen identifies a discrete region of the FXR2P LC domain as a regulator of assemblage formation in neurons.** (A) Schematic of EGFP insertional mutagenesis approach used to generate a set of 18 unique, full-length EGFP-transposed FXR2P constructs (FXR2P^EGFP^; see Materials and Methods). Each green tag marks one specific EGFP insertion site within a FXR2P^EGFP^ construct. EGFP insertion at a given residue is denoted as FXR2P^[#]^ where ‘#’ is the amino acid insertion position in FXR2P. (B–D) Cultured cortical neuron co-transfected with FXR2P^[217]^ (green) and diffusible cell fill TdTomato (red). FXR2P^[217]^ is present diffusely in the soma (C) and in granules in dendrites (D). (E–G) FXR2P^[416]^ is present in Type A fibril bundles in both soma (F) and dendritic processes (G). (H–J) FXR2P^[435]^ is present in dendritic granules (arrows) as well as Type B fibril bundles within soma (I) and dendritic processes (J). (K–M) FXR2P^[456]^ is present in dendritic granules; no fibrils were observed in either soma (L) or processes (M). All cultures are DIV14. Representative images from *n*=3 experiments. Scale bars: 20 µm. Tud, Tudor domain; NLS, nuclear localization signal; KH1, RNA-binding KH1 domain; KH2, RNA-binding KH2 domain; NES, nuclear export sequence; NOS, nucleolar targeting signal.

We performed an EGFP insertional mutagenesis screen to reveal intrinsic regions of FXR2P that might influence its higher-order structural state. We anticipated that this approach could yield at least two broad classes of FXR2P^EGFP^ fusions that might: (1) mimic the ability of FXR2P to assemble into the typical granule structure observed endogenously in neurons or (2) promote or disrupt the formation of higher-order assemblages. A library of FXR2P^EGFP^ insertional constructs was created using an *in vitro* transposition reaction that capitalizes on transposons to introduce EGFP coding sequence (CDS) into single sites within FXR2P (Fig. 1A; Sheridan et al., 2002; Giraldez et al., 2005). We first isolated constructs that had EGFP insert in-frame and in the proper orientation by expressing the library in *Escherichia coli* and selecting fluorescent colonies. Of the 180 selected constructs, 18 had EGFP inserted into a unique position along the length of FXR2P CDS (Table 1). As shown in Fig. 1A and Table 1, we observed that EGFP insertions were obtained throughout the majority of the FXR2P coding sequence. Thus, this procedure yielded 18 different fusions that we term ‘FXR2P^[X]^’ where X is the amino acid position at which EGFP is inserted (Table 1, Fig. 1A).

**Table 1.**
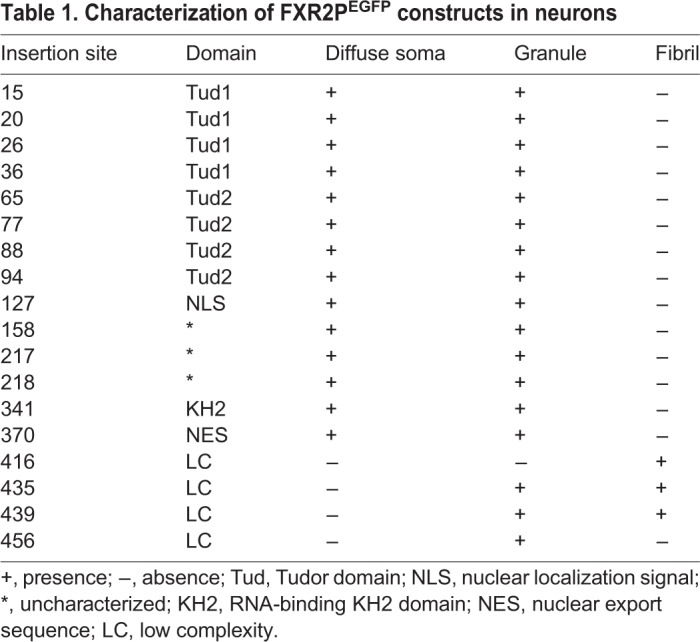
**Characterization of FXR2P^EGFP^ constructs in neurons**

### EGFP fusions in the amino terminal half of FXR2P form granules localized to neuronal processes

To determine which domains of FXR2P influence its formation into higher-order assemblages we assessed the cellular distribution of the 18 FXR2P^EGFP^ fusions expressed in cultured neurons (Fig. 1A, Table 1). Primary cortical neuron cultures were co-transfected at DIV3 (days *in vitro*) with each of the FXR2P^EGFP^ constructs along with TdTomato to provide a diffusible cell fill. As summarized in Table 1, fusions with EGFP inserted into any of 14 different sites in the N-terminal half of FXR2P were diffusely distributed in cell somata and localized to discrete granules within dendrites and axons of DIV6 and DIV14 neurons (Fig. 1B–D). This pattern was observed with EGFP fused into one of several regions within the N-terminal half of the molecule including the Tudor, RNA-binding KH2 and nuclear localization signal domains (Fig. 1A, Table 1). FXR2P^[217]^ was chosen as the representative of this set of 14 N-terminal fusions (Fig. 1B–D). The cellular distribution of FXR2P^[217]^ is qualitatively similar to that previously observed for heterologously-expressed FXR2P (Levenga et al., 2009; see also Stackpole et al., 2014) and for endogenous FXR2P (Fig. S1). Taken together, these data indicate that FXR2P with EGFP inserted within N-terminal domains organizes into granular assemblages within neurons.

### EGFP insertion within a restricted region of the LC domain results in the formation of two distinct fibrillar states of FXR2P^EGFP^

**Fig. 2.**
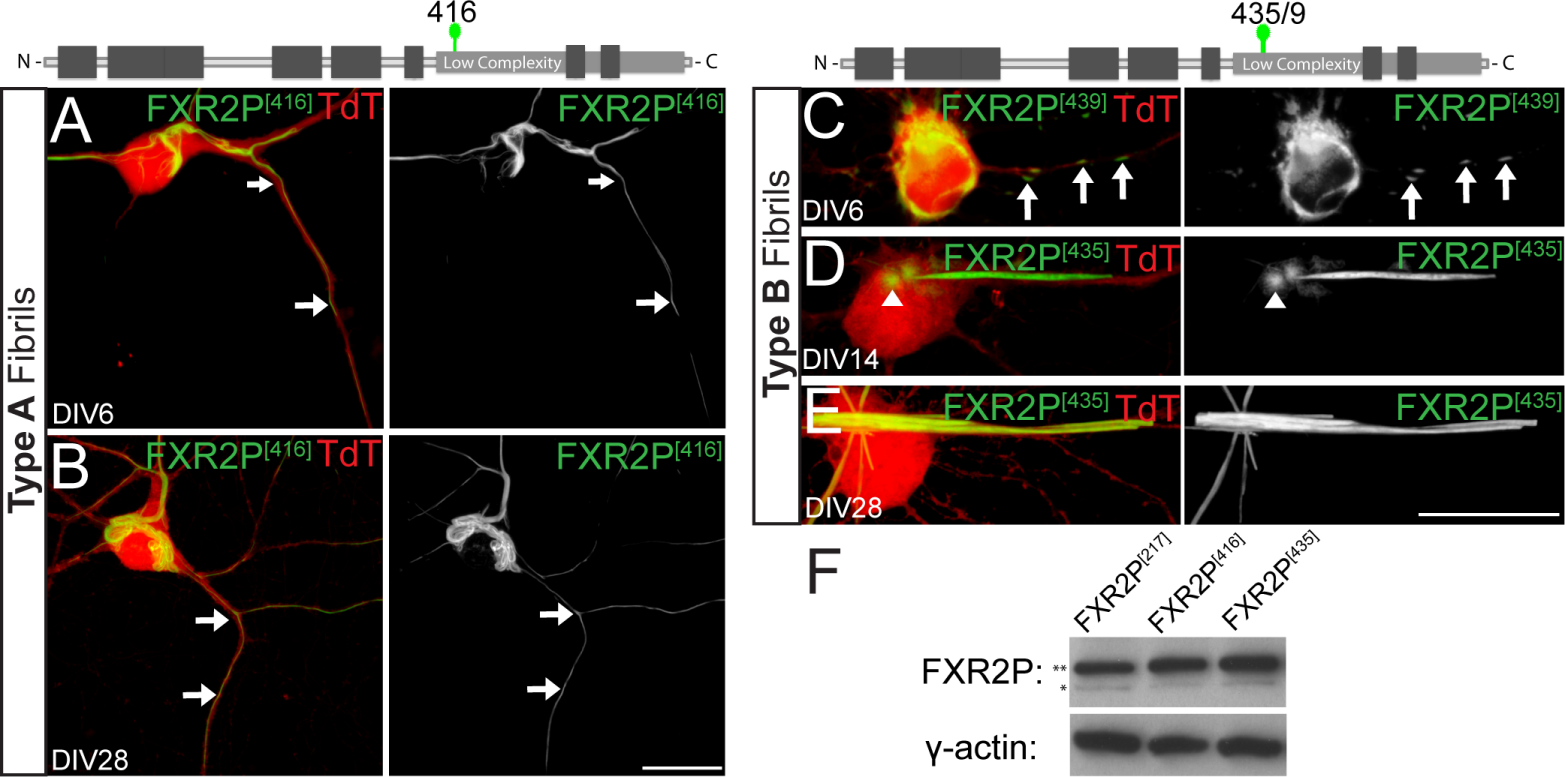
**Time course of Type A and B fibril bundle expression in cultured neurons.** (A) DIV6 neuron co-transfected with FXR2P^[416]^ (green) and TdTomato (red). Type A fibril bundles are detected in the cell body and extending into dendritic processes. Arrows mark localization of fibrils in dendrites. Dendritic FXR2P granules are not observed. (B) Type A fibril bundles observed in soma and dendrites of a DIV28 neuron. Arrows mark FXR2P fibrils in a dendrite. (C) Dendrite of a DIV6 neuron co-expressing FXR2P^[439]^ (green) and TdTomato (red). FXR2P^[439]^ is present in dendrites only in granules at this time (arrows). (D) In DIV14 neurons, FXR2P^[435]^ localizes to discrete, spherical, nest-like structures with a fibrillar substructure (arrowhead). These nest-like structures are closely associated to newly forming Type B fibril bundles. (E) FXR2P^[435]^ forms Type B fibril bundles in a DIV28 neuron that extend from the cell body into dendritic processes. (F) Western blot analysis of protein lysates from DIV6 neuronal cultures expressing FXR2P^[217]^, FXR2P^[416]^ or FXR2P^[435]^ probed with antibodies to FXR2P and γ-actin (loading control; ∼43kD). Note that all the FXR2P^[EGFP]^ fusions are expressed at similar levels. Equivalent results were observed in two independent experiments. The anti-FXR2P detected both the FXR2P^[EGFP]^ fusions (upper band ∼122kD; double asterisk) as well as endogenous FXR2P (lower band ∼95kD; single asterisk). Representative images from *n*=3 experiments for DIV6 and DIV14; *n*=2 for DIV28. Scale bars: 20 µm.

EGFP fused within a restricted region of the FXR2P C-terminal LC domain assumed strikingly different organizations when expressed in neurons. When viewed at the light microscopic level, FXR2P^EGFP^ fusions at residue 416 (FXR2P^[416]^) assembled into elongated, curvilinear structures that we term Type A fibril bundles (Fig. 1E–G; see ultrastructural analysis below). Type A fibril bundles were thread-like, apparently flexible structures present in somata, axons and dendrites. Neurons containing Type A fibril bundles exhibited few to no FXR2P^[416]^ granules within processes. Moreover, little diffuse signal was observed anywhere in the cell, suggesting that the large majority of FXR2P^[416]^ had assembled into the fibril bundles. FXR2P^[416]^-expressing neurons exhibited Type A fibril bundles at all times examined (DIV6–28; Fig. S2), with their length and complexity increasing with age (Fig. 2A,B; Fig. S2). Together, these data demonstrate that FXR2P^[416]^ adopts a distinctive fibrillar organization across multiple developmental stages in cultured neurons.

FXR2P^EGFP^ fusions at either residue 435 or 439 in the LC domain assembled into a second remarkable structure when expressed in neurons that we term Type B fibril bundles (Figs 1H–J and 2C–E, also see below). Since fusions at either of these two positions yielded similar results (Fig. S2), we will use the term ‘FXR2P^[435/439]^’ to describe their properties; specific constructs used for given experiments are noted in the Figures. Type B fibril bundles were straight and crystal-like structures. These bundles were observed at ≥DIV11 and were present in both somata and dendrites (Fig. 2C–E). Type B fibril bundles were observed for up to 4 weeks in culture with bundle size increasing over time (Fig. 2C–E; Fig. S3). FXR2P^[435/439]^ was also localized to smaller dendritic granules at all time points investigated (Figs 1 and 2). FXR2P^[435/439]^ was also present within spherical, nest-like, finely fibrous structures that were closely associated with Type B fibril bundles (Fig. 2D). These nest-like structures, reminiscent of dandelion seed pods, were only observed from ∼DIV9–14. Finally, FXR2P fusions at residue 456 within the LC domain (FXR2P^[456]^) formed neither fibrillar nor nest-like structures in neurons. Rather, FXR2P^[456]^ was localized to granules that were widely distributed within the cell somata and neuronal processes (Fig. 1K–M), similar to that observed with FXR2P^[217]^. Thus, EGFP insertion into a discrete region within the LC domain (residues 416–439) resulted in two novel types of FXR2P^EGFP^ fibrillar structures when expressed in neurons.

We considered the possibility that the fibrillar bundles formed by FXR2P^[416]^ and FXR2P^[435/439]^ might be caused by differential expression of these fusions and/or reflect reduced cell viability. However, western blotting demonstrated that all the fusions tested showed equivalent FXR2P^EGFP^ expression levels (Fig. 2G). Moreover, this biochemical analysis indicated that all FXR2P^EGFP^ fusions tested were intact, with no signs of cleavage. Further, as judged by the TdTomato cell fill, neurons expressing any of the FXR2P^EGFP^ fusions demonstrated comparable somatodendritic and axonal morphologies (Fig. 1) for up to 4 weeks in culture (Fig. 2). Taken together, these observations suggest that Type A and B fibril bundles most likely form due to intrinsic differences in protein assembly caused by EGFP insertion into the LC domain.

We also questioned whether EGFP insertion in the LC domain of FXR2P caused a similar fibrillization effect in non-neuronal cells. We therefore transfected mammalian COS-7 cells with each FXR2P^EGFP^ construct and observed their localization after 24 h. In comparison to endogenous FXR2P and FXR2P^[217]^ that distribute diffusely throughout the cytoplasm (Fig. S3A,B), the FXR2P^EGFP^ fusions in the LC domain instead showed an amorphous, perinuclear distribution (Fig. S3C,D). However, no FXR2P fibrils were observed in COS-7 cells, suggesting that FXR2P^EGFP^ fibrillization is specific to neurons.

### FXR2P is an intrinsically disordered protein with a non-prion-like LC domain

**Fig. 3.**
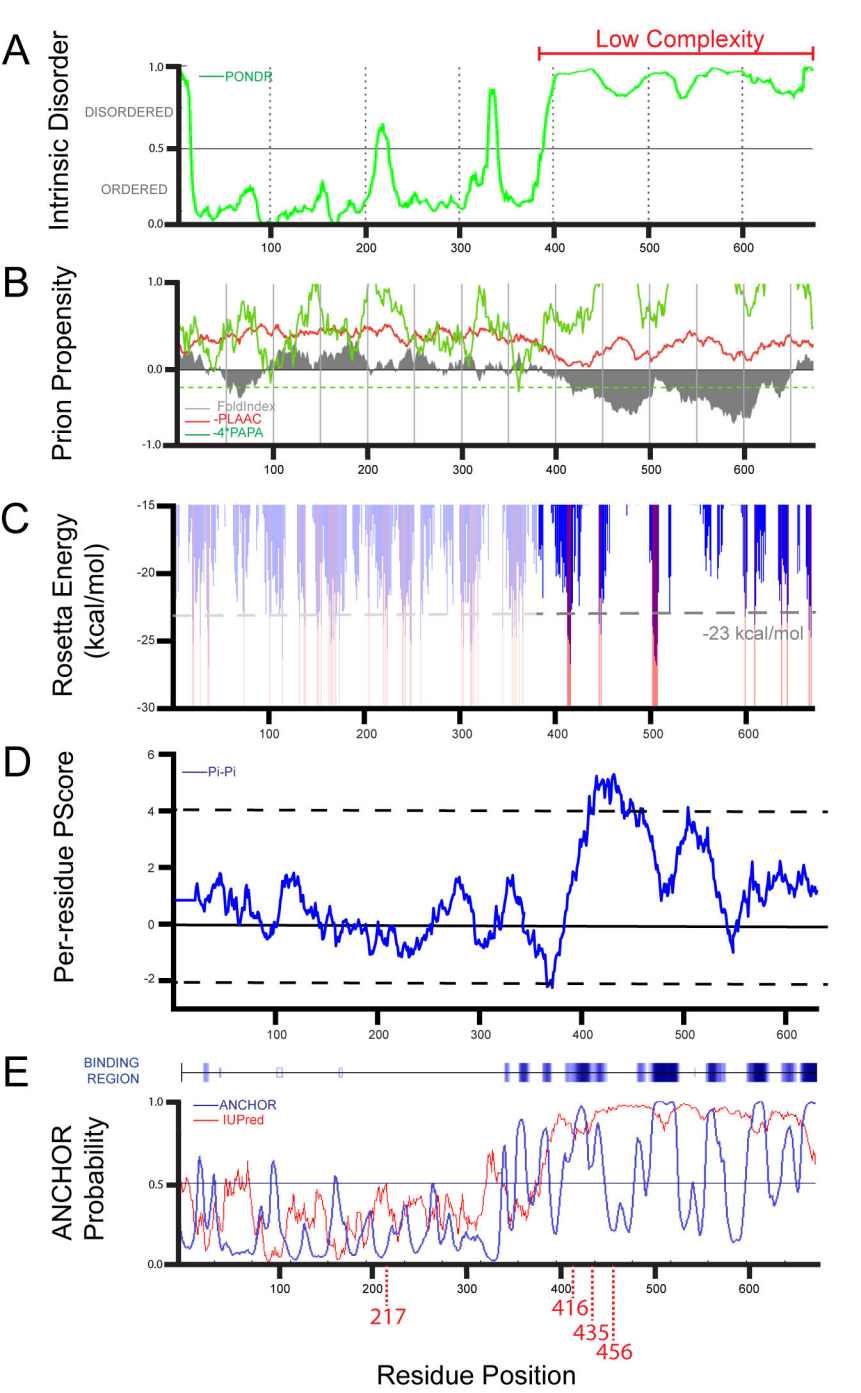
**FXR2P contains a C-terminal LC domain that is intrinsically disordered.** (A) PONDR-FIT predicts that a low complexity region in the C-terminus of FXR2P (residues 388–673) is intrinsically disordered. PONDR-FIT scores > 0.5 indicate a probability towards intrinsic disorder. (B) FoldIndex predicts that the LC region of FXR2P is intrinsically unfolded (gray shading shows less than zero). However, no region within FXR2P was predicted to be prion-like by either the PLAAC algorithm (red, log-likelihood ratio score below zero) or the PAPA algorithm (green, log-odds ratio score below dashed green line). (C) ZipperDB predicts that regions around residues 415 and 500 have increased fibrillization propensity. [Note that the apparent increased fibril-forming propensity of regions N-terminal to the low complexity domain (light shading) are due to the folded nature of these domains and are not relevant to this analysis.] (D) Phase separation prediction based on the per-residue pi-contact propensity indicates that the low complexity region of FXR2P has increased propensity to form pi-contacts compared to its folded domains. Dotted lines represent PScore thresholds for enrichment of pi-contacts (PScore ≥ 4) or depletion of pi-contacts (PScore ≤ −2). (E) ANCHOR predicts multiple disordered binding regions within the LC domain of FXR2P (blue, score > 0.5; darker blue signifies higher ANCHOR score). IUPRED predicts the LC domain as intrinsically disordered segment (red, score >  0.5 are predicted as disordered). Red lines below the residue position denote the location of the 217, 416, 435/439 and 456 EGFP insertion sites.

The N-terminal half of FXR2P shares high sequence similarity and domain organization with FMRP, an extensively characterized protein (Zhang et al., 1995; Darnell et al., 2009; Adams-Cioaba et al., 2010). In contrast, little is known about the C-terminal half of FXR2P. We therefore analyzed FXR2P using the bioinformatic predictors of intrinsically unfolded and disordered regions PONDR-FIT and FoldIndex (Xue et al., 2010; Prilusky et al., 2005). Both algorithms predicted that the C-terminal half of FXR2P harbors an LC domain with an intrinsically disordered and unfolded structure (residues ∼388–673; Fig. 3A,B). We next determined whether the FXR2P LC domain was predicted to harbor the intrinsic ability to fibrillize into steric zipper structures. ZipperDB (Goldschmidt et al., 2010) predicted multiple residues in the LC domain with a high propensity for fibrillization (Fig. 3C). Interestingly, one of these regions, residues 415–419 (ESSSS; Fig. 3C) was coincident with the EGFP insertion site that results in Type A fibril formation (FXR2P^[416]^, see above). A high contribution of residues prone to pi–pi contact formation (Fig. 3D) suggests that FXR2P may self-interact like other pi-rich proteins that undergo phase separation, a critical regulatory feature of LC-driven self-assembly and organization of higher-order macromolecules (Mitrea and Kriwacki, 2016; Chong and Forman-Kay, 2016; Vernon et al., 2018). Taken together with the ZipperDB analyses, the LC domain of FXR2P is thus predicted to be primed for phase separation and/or intrinsic self-assembly.

We also asked whether the FXR2P LC domain contained prion-like elements, which are characteristic of RBPs implicated in neurodegenerative disease (Harrison and Shorter, 2017; King, Gitler and Shorter, 2012). However, Fig. 3B shows that the FXR2P LC domain lacks Q/N rich regions as judged by either the PAPA or PLAAC algorithms (Toombs et al., 2012; Lancaster et al., 2014). Finally, we used ANCHOR to predict the locations of disordered binding regions within the FXR2P LC domain (Dosztányi et al., 2009). This algorithm failed to detect favorable intrachain interactions in the LC domain that might promote folding into a well-defined structure, but did identify disordered protein segments that could undergo a disorder-to-order transition upon binding with globular protein partners (Fig. 3E; Mészáros et al., 2009). Notably, such predicted *trans* domains were present within the discrete region targeted in the fusion constructs (positions 416–439), but were absent from the position 456 region (Fig. 3E). Together, these algorithms indicate that FXR2P is an intrinsically disordered protein containing a large, non-prion-like LC region constituting ∼42% of its length and has the potential to restructure into defined three-dimensional shapes upon protein binding.

### FXR2P^EGFP^ LC fusions form distinct fibril types that assemble into bundles with differential ribosome association

**Fig. 4.**
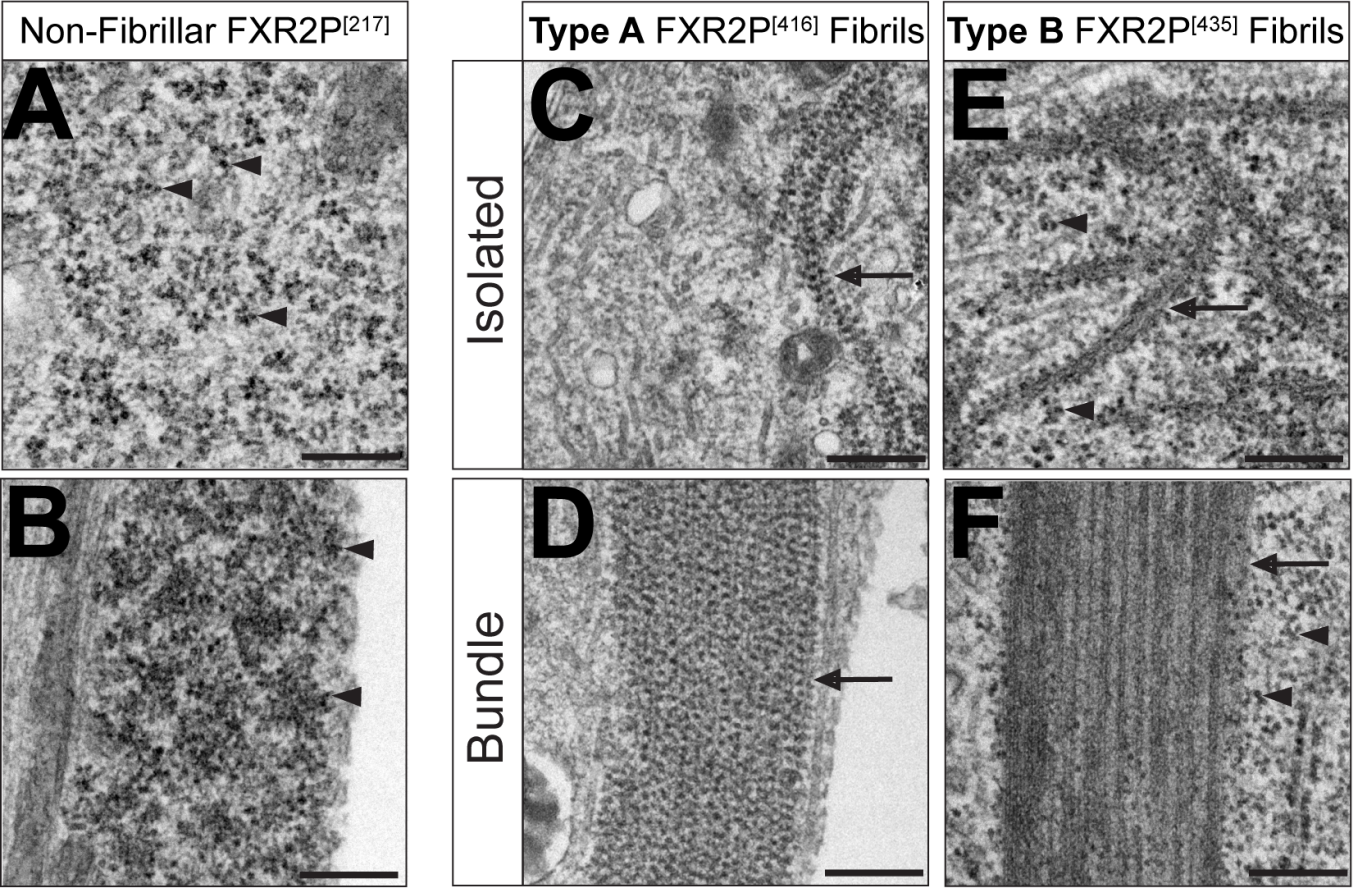
**Ultrastructure of FXR2P^[EGFP]^ Type A and B isolated fibrils and fibril bundles in neurons.** Electron microscopy of neurons transfected with non-fibrillar FXR2P^[217]^ (A,B); Type A fibril-forming FXR2P^[416]^ (C,D); or Type B fibril-forming FXR2P^[435]^ constructs (E,F). (A) Cytoplasm of DIV6 neuron expressing FXR2P^[217]^ is rich in polysomes (arrowheads) and no fibrils are observed. (B) Ribosome (arrowhead)-enriched dendritic granule in a neuron expressing FXR2P^[217]^. (C) Isolated Type A fibrils decorated with ribosomes (arrow) from DIV6 neuron transfected with FXR2P^[416]^. Note that polyribosomes are not observed in the cytoplasm. (D) Type A fibril bundle (arrow) associated with ribosomes in a neuron expressing FXR2P^[416]^. (E) DIV14 neuron transfected with FXR2P^[435]^ displays ultrastructurally distinct, isolated Type B fibrils decorated with ribosomes (arrows). Polyribosomes are observed in the cytoplasm (arrowheads). (F) Type B fibril bundle (arrow) devoid of ribosomes in a neuron expressing FXR2P^[435]^. Note that polysomes are readily observed in the adjacent cytosol (arrowheads). Representative images from *n*=3 neurons per condition. Scale bars: 250 nm.

We next investigated the ultrastructure of neurons expressing FXR2P^[217]^, FXR2P^[416]^ or FXR2P^[435]^ (granules only, Type A fibrils, or Type B fibrils, respectively; see above). Neurons expressing FXR2P^[217]^ contained numerous polysomes in somata (Fig. 4A) and discrete structures within dendrites that were rich in ribosomes embedded in electron-dense, amorphous material (Fig. 4B). These structures are likely to correspond to the granules observed by light microscopy (Fig. 1). No fibrillar structures were observed in cells expressing FXR2P^[217]^.

**Fig. 5.**
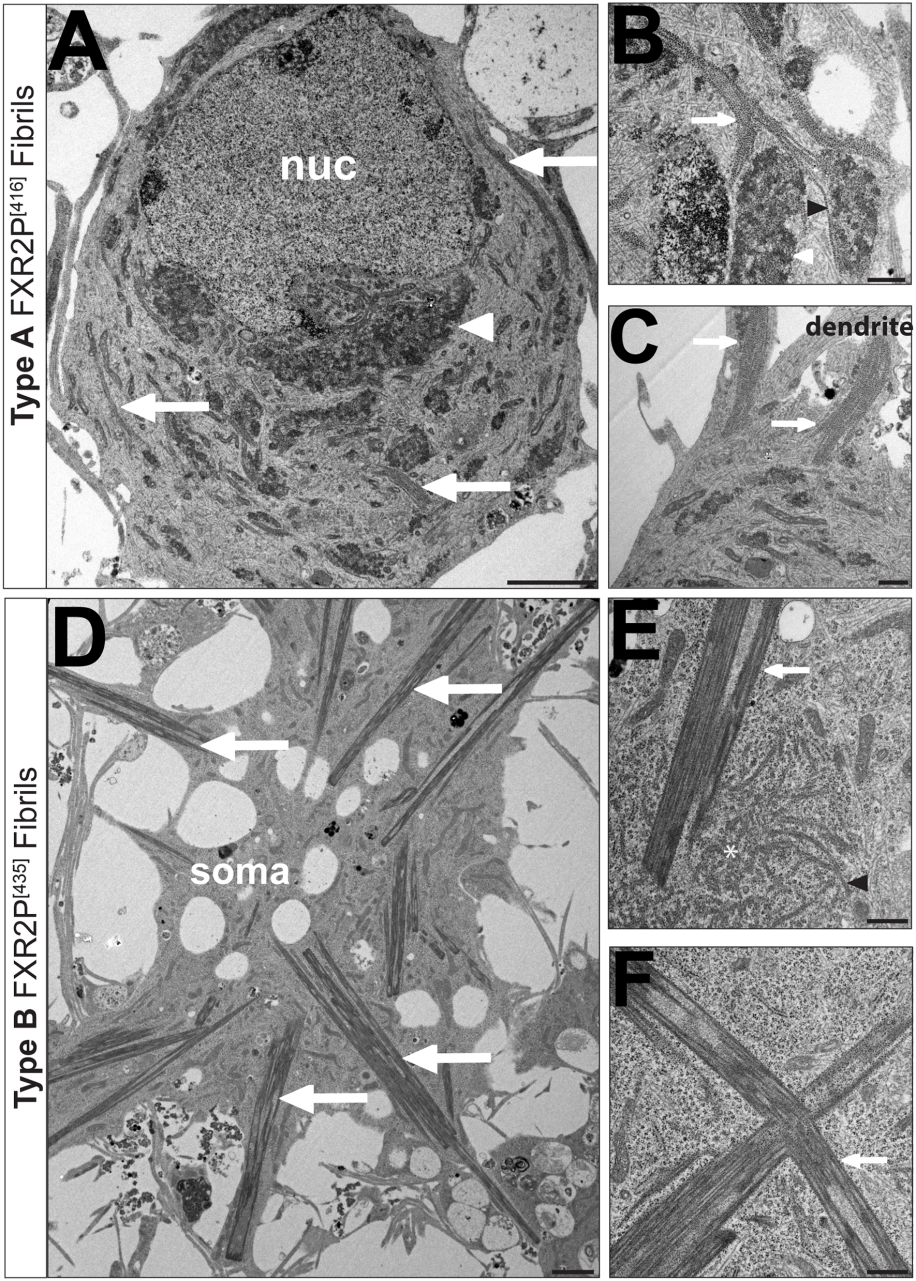
**Disposition and ultrastructural localization of Type A and B FXR2P isolated fibrils and fibril bundles in neurons.** (A–C) Electron micrographs of DIV6 cortical neurons transfected with Type A fibril-forming FXR2P^[416]^. (A) Low-magnification view of cell soma shows Type A fibril bundles coursing through cytoplasm (arrows). Juxtanuclear amorphous material is also observed (white arrowhead). (B) Isolated Type A fibrils (black arrowhead) and bundles (arrow) in close proximity to granular material (white arrowhead). Note the apposition of the isolated Type A fibril with the granular domain. (C) Type A fibril bundles (arrows) extend from cell soma into dendritic processes. (D–F) Electron micrograph of DIV14 cortical neurons expressing Type B fibril-forming FXR2P^[435]^. (D) Low-magnification view of cell shows multiple rigid Type B fibrils extending radially from cell center (arrows). Plane of section is adjacent to the substrate. (E) Isolated Type B ribosome-decorated fibrils (black arrowhead) are restricted to a circular domain in the cytoplasm (white asterisk), which is likely to be a section of the nest-like structures observed by fluorescence microscopy (Fig. 2D). Note that a ribosome-free Type B fibril bundle (white arrow) is juxtaposed to this nest-like structure. (F) Type B fibril bundles (white arrow) are rectilinear and jagged in the cytoplasm as well as devoid of ribosomes. Representative images from *n*=3 neurons per condition. Scale bars: 2 µm (A,B), 500 nm (B,C,E,F). nuc, nucleus.

Electron microscopy of neurons expressing FXR2P^[416]^ revealed distinctive cytoplasmic fibrils that were present in both isolated and bundled configurations (Figs 4C,D and 5A–C). Isolated Type A fibrils were strand-like and thin (width: 20.5±0.7 nm; mean±s.e.m.; *n*=9 fibrils, 3 neurons). These strand-like structures were observed coursing within the cytoplasm of cell bodies (Fig. 4C) and were frequently adjacent to perinuclear amorphous, electron-dense material (Fig. 5A,B). This co-localization suggests that this electron-dense material could act as a zone for fibril production.

Type A fibrils were also present in highly organized bundles (Figs 4D and 5B,C). These fibril bundles were wide structures (Fig. 4D) observed throughout the somata and extending into dendrites (Fig. 5A–C). The size and disposition of these fibril bundles indicate that they correspond to the thread-like structures observed by fluorescence microscopy (Figs 1 and 2). Taken together, these observations indicate that isolated FXR2P^EGFP^ Type A fibrils assemble into bundles with no variation in their structure.

Ultrastructural analysis of neurons expressing Type B fibril-forming FXR2P^[435]^ revealed a strikingly different class of isolated fibrils and fibril bundles. Isolated Type B fibrils were short and arc-like (average width: 41.6±1.5 nm; *n*=12 fibrils, 2 neurons; Fig. 4E) and clustered within discrete spherical zones (Fig. 5E). These zones are likely to correspond to the spherical, nest-like structures adjacent to Type B fibril bundles observed by fluorescence microscopy (Fig. 2D).

Neurons expressing FXR2P^[435]^ also exhibited highly regular fibril bundles with an overall needle-like appearance as they coursed through the cytoplasm (Figs 4F and 5D–F). Type B bundles extended radially from cell somata into processes (Fig. 5D) and were comprised of tightly aligned individual fibrils (Fig. 4F). This observation suggests that isolated Type B fibrils may transition from a short, arc-like state to a rigid, elongated configuration when assembled into the Type B bundles. Finally, the clusters of isolated Type B fibrils were often juxtaposed to fibril bundles (Fig. 5E), suggesting that these zones may be organizing centers for fibril bundling.

### Type A and B FXR2P^EGFP^ fibril bundles show differential ribosome association

We next assessed the ribosomal association of the FXR2P^EGFP^ fibrils. Fig. 4 shows that ribosomes associated with both isolated and bundled Type A fibrils (FXR2P^[416]^) in a periodic fashion with a spacing of 28.9±1.5 nm and 31.4±0.7 nm, respectively (*n*=10 and 14 fibrils, respectively, 3 neurons; Fig. 4C,D). Remarkably, electron microscopy also revealed the wholesale redistribution of ribosomes within cells expressing FXR2P^[416]^. In contrast to neurons expressing FXR2P^[217]^, where polysomes were widely distributed in the cell somata (Fig. 4A), neurons expressing FXR2P^[416]^ showed ribosomes decorating Type A fibrils and were not observed free in the cytosol (Fig. 4C,D).

Isolated and bundled Type B fibrils showed strikingly differential ribosome association. Although ribosomes associated with isolated Type B fibrils (Fig. 4E), Type B fibril bundles were devoid of ribosomes (Fig. 4F). Further, ribosomes were abundant in the cytosol of neurons containing Type B fibrils (Figs 4F and 5E,F). Taken together, these results show that insertion of EGFP into specific sites within the FXR2P LC domain results in the formation of two structurally distinct, ribosome-decorated isolated fibril types (A and B). However, when assembled into bundles, FXR2P^EGFP^ fibrils either associate with (Type A) or are devoid of (Type B) ribosomes. Further, virtually all ribosomes in neurons expressing FXR2P^[416]^ partition to Type A fibrils.

**Fig. 6.**
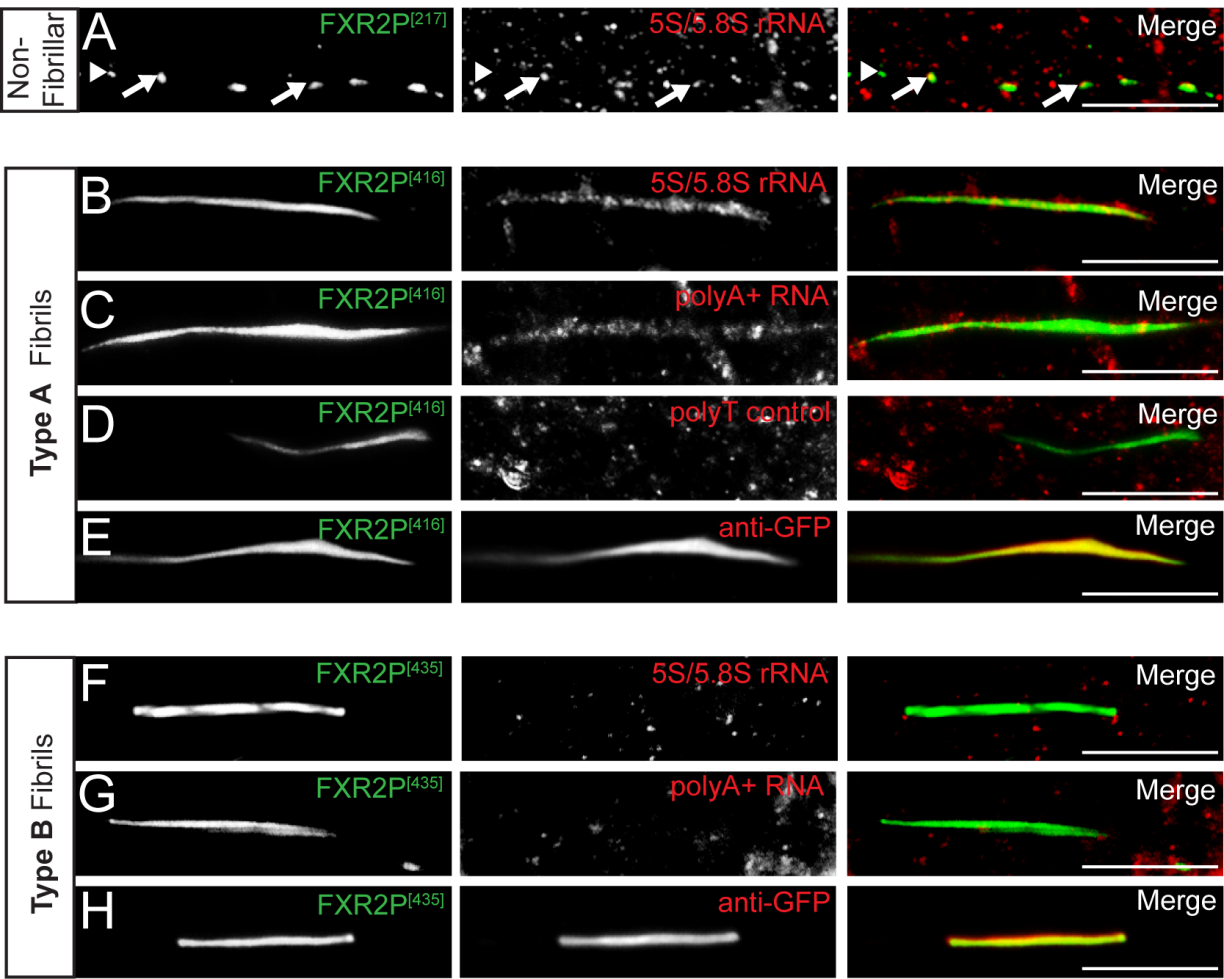
**Ribosomes and mRNA co-localize with Type A but not Type B fibril bundles.** (A) A subset of FXR2P^[217]^ (green) granules colocalize (arrows) with 5S/5.8S rRNA (Y10b antibody; red) in dendrites. Arrowheads denote FXR2P^[217]^-only granules. (B–E) Type A fibrils (green) colocalize with 5S/5.8S rRNA (red; B) and polyA+ mRNA (red; C) in dendrites. No signal was observed in fibrils with a polyT control (red; D). Anti-GFP immunostaining (red) of Type A fibrils (green; E). Note the complete co-localization of FXR2P^[416]^ intrinsic EGFP signals with the anti-GFP immunostain. (F–H) Type B fibrils (green) do not colocalize with either 5S/5.8S rRNA (red; F) or polyA^+^ mRNA (red; G). Intrinsic GFP signal of FXR2P^[435]^ Type B fibril bundles co-localize with the anti-GFP immunostain (red; H). Representative images from *n*=2–3 experiments. DIV14 neurons. Scale bars: 10 µm.

We used fluorescent microscopy to confirm and extend the ultrastructural observations of ribosome association with FXR2P fibrils. We first asked whether FXR2P^[217]^ colocalized with either rRNA in neurons. Using a monoclonal antibody that recognizes 5S/5.8S rRNA (Y10b; see Materials and Methods), we observed that only a subset of FXR2P^[217]^ colocalized with rRNA within dendritic granules of DIV14 neurons (Fig. 6A). Colocalization was also observed for Type A fibril bundles with 5S/5.8S rRNA and polyA^+^ RNA (oligodT probe; Fig. 6B,C). No signal was observed when sense probes were used (Fig. 6D).

Finally, in agreement with our ultrastructural analyses, no rRNA or polyA^+^ RNA was detected in Type B fibril bundles (Fig. 6F,G). We considered the possibility that the rigid and tightly ordered structure of Type B fibrils might restrict antibody accessibility. To address this potential confound, we performed immunofluorescence with an anti-GFP antibody and observed that the signal from this reagent was indistinguishable from that of the respective FXR2P^EGFP^ fusions (Fig. 6E,H). These findings provide further evidence that manipulation of the FXR2P LC domain results in the assembly of two distinct higher-order states in neurons distinguishable by their structure and ability to associate with RNA.

### Both RNA-binding and LC domains are required for FXR2P^EGFP^ fibril formation in neurons

**Fig. 7.**
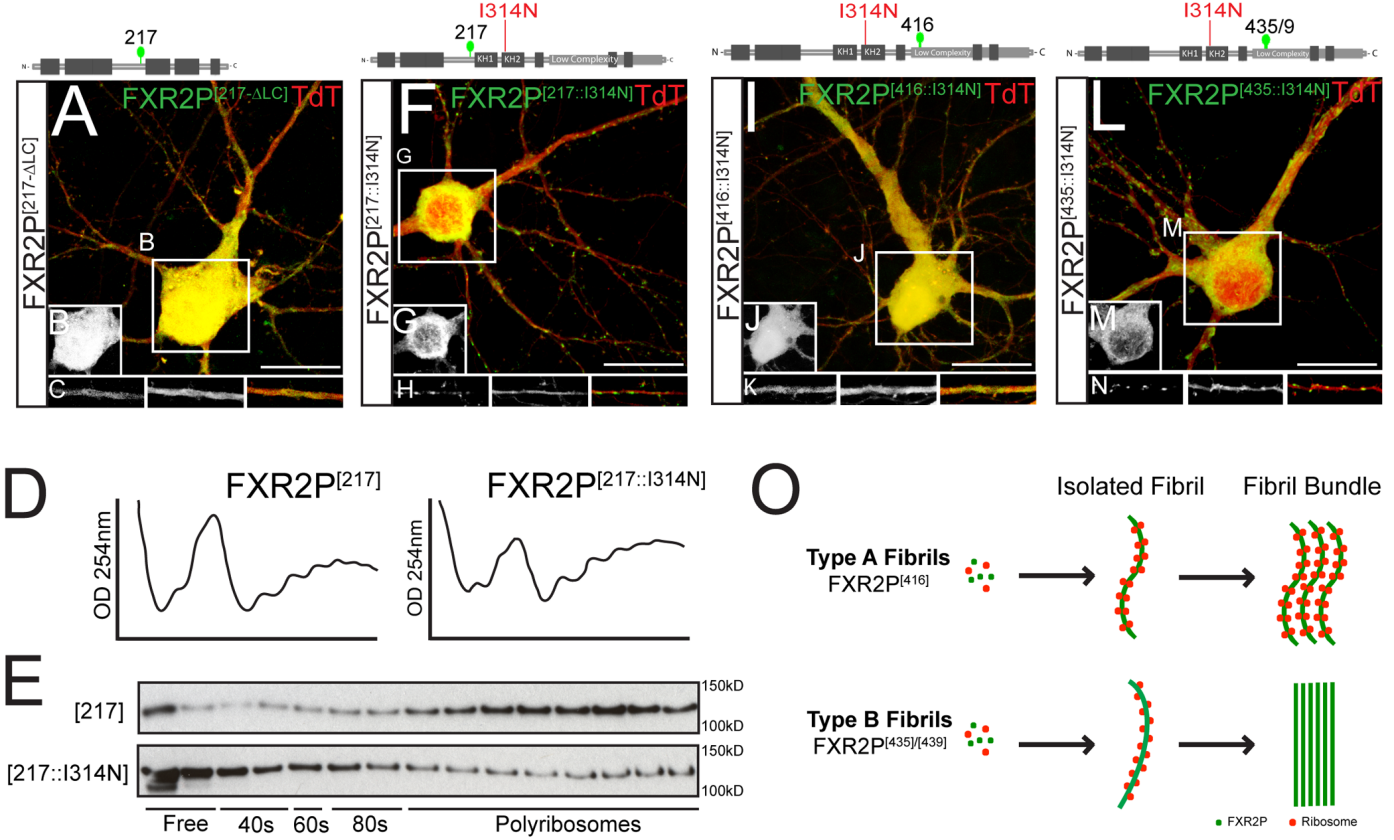
**FXR2P RNA-binding and LC domains collaborate in fibril formation.** (A–C) DIV14 neuron co-transfected with (FXR2P^[217ΔLC]^; green) and TdTomato (red). FXR2P^[217ΔLC]^ is diffusely localized throughout the cell and discrete granules are not observed (B and C, respectively). (D) A_254_ traces of sucrose gradients from HEK293T cells expressing either FXR2P^[217]^ or the RNA-binding mutant FXR2P^[217::I314N]^. (E) Western blot for FXR2P in fractions collected from sucrose gradients from either FXR2P^[217]^ (upper blot) or FXR2P^[217::I314N]^ (lower blot). FXR2P^[217]^ predominantly co-sediments in polysome fractions. In contrast, FXR2P^[217::I314N]^ is enriched in free and monosome fractions and present in low levels in polysome fraction. Equivalent results were observed in two independent experiments. (F–H) FXR2P^[217::I314N]^ (green) contains a point mutation in the RNA-binding KH2 domain and is diffusely localized in the soma (G) and present in granules in cell processes (H). Compare to Fig. 1B–D. (I–K) FXR2P^[416::I314N]^ (green) is expressed diffusely in the nucleus and cell processes (J and K, respectively). No granular or fibrillar structures are observed. Compare to Fig. 1E–G. (L–N) FXR2P^[435::I314N]^ (green) is diffusely distributed in the soma (M) and is present in granules in dendrites (N). No fibrillar structures are observed. Compare to Fig. 1H–J. (O) Summary of Type A and B fibril dynamics and ribosome association in neurons. See text for details. Representative images of *n*=2–3 experiments. Neurons transfected at DIV3 and analyzed on DIV14. Scale bars: 20 µm.

We next asked whether the LC domain is necessary for FXR2P^EGFP^ assemblage formation in neurons. As shown in Fig. 7, a mutant FXR2P lacking the LC domain (FXR2P^[217ΔLC]^) was distributed diffusely throughout the cell somata and processes. Neither granules nor fibrils were observed (Fig. 7A–C). Combined with the above results, these data indicate that the LC domain is required for its ability to form higher-order FXR2P^EGFP^ assemblages within neurons.

We then sought to investigate the role of FXR2P RNA binding in assemblage formation. Mutations that abrogate FXR2P RNA binding have not been reported. However, the FXR2P KH2 domain is over 90% identical to that in FMRP (Zhang et al., 1995). Moreover, an I304N point mutation in the FMRP KH2 domain abrogates its RNA binding and ribosome association (Zhang et al., 1995; Feng et al., 1997; Laggerbauer et al., 2001; Darnell et al., 2005a,b; Zang et al., 2009; Ascano et al., 2012). We therefore mutated the comparable residue in FXR2P (I314N). To directly test whether this I314N mutation affects polysome association, we analyzed ribosome co-sedimentation by sucrose gradients from extracts of HEK293T cells expressing either FXR2P^[217]^ or FXR2P^[217::I314N]^. Fig. 7D,E shows that FXR2P^[217]^ was enriched in polysome fractions. This sedimentation profile of FXR2P^EGFP^ is in agreement with previous work demonstrating that endogenous FXR2P predominantly co-fractionates with polysomes (Siomi et al., 1996; Feng et al., 1997; Darnell et al., 2009; Zang et al., 2009). In contrast, FXR2P^[217::I314N]^ was shifted to lighter, non-polysome fractions (Fig. 7E). The A_254_ traces were similar between FXR2P^[217]^ and FXR2P^[217::I314N]^ extracts, indicating that global translation was similar in cells expressing either FXR2P form (Fig. 7D). Together, these results indicate that the I314N mutation perturbs FXR2P^EGFP^ RNA binding.

We then assessed the role of FXR2P^EGFP^ RNA binding in neuronal assemblage formation. As shown in Fig. 7F–H, FXR2P^[217::I314N]^ was distributed diffusely in cell bodies and was localized to granules in dendrites, a comparable localization to that observed with FXR2P^[217]^ (see Fig. 1). Thus an intact LC domain, but not RNA binding ability, is required for the formation of granular FXR2P^EGFP^ assemblages in neurons. In contrast, the RNA-binding mutant FXR2P^[416::I314N]^ was diffusely distributed in the somata and cell processes; neither fibril bundles nor granular assemblages were observed (Fig. 7I–K). Finally, FXR2P^[435::I314N]^ was present in granular assemblages within the neuronal processes and was localized diffusely in the somata. No fibrillar structures were observed, even after DIV14 (Fig. 7L–N). These compact dendritic granules were similar to those observed in neurons expressing FXR2P^[435/9]^ at DIV6–28 (See Fig. 2C). Moreover, the fibrous, nest-like structures observed at >DIV9 were not detected (Fig. 7L–N). Taken together, these data indicate that collaboration between distinct regions within the LC and the RNA-binding domain are required to drive formation of both Type A and Type B FXR2P^EGFP^ fibril bundles in neurons.

## DISCUSSION

In this study, we demonstrate that the higher-order structural state of FXR2P is influenced by manipulation of its low complexity and RNA-binding domains. We first defined these regions using an insertional mutagenesis approach, revealing that EGFP insertion at discrete sites within the LC domain impacted FXR2P^EGFP^ higher-order structural states. FXR2P^EGFP^ LC mutants can assume multiple higher-order states with unique ultrastructures, developmental timelines and ribosome/RNA association in neurons (Fig. 7O). Our data suggest that these states are largely formed due to intrinsic differences in protein assembly caused by EGFP insertion at specific sites within the LC domain, but also require a functional KH2 RNA-binding domain. Our results indicate an important role of distinct LC regions in regulating the formation of RBP complexes in a neuronal context and suggest potential mechanisms by which different structural and higher-order states of an RBP can influence its protein and RNA association.

We utilized an EGFP insertional mutagenesis approach to identify FXR2P domains that influence its assembly into higher-order complexes. Several observations indicate that the formation of these distinct assemblages requires EGFP insertion at specific sites in the LC domain along with an intact KH2 RNA-binding domain. First, of the 18 EGFP fusions studied, 15 had no detectable fibrillar organization when expressed in neurons (Table 1). Instead, fibrillar FXR2P^EGFP^ was observed in only 3 fusions that harbored insertions within a 23 amino acid stretch of the LC domain (residues 416–439; Figs 1 and 3). Second, an FXR2P fusion with EGFP inserted at position 456 in the LC domain (Fig. 1) did not assemble into fibrils. Third, as discussed below, FXR2P^[416]^ or FXR2P^[435]^ only formed fibrils when the KH2 RNA-binding domain, which is in the central region of the protein, was functionally intact (Fig. 7). Fourth, deletion of the LC domain resulted in the complete loss of FXR2P assemblages in neurons (FXR2P^[217ΔLC]^; Fig. 7). Moreover, the formation of these assemblages was not secondary to expression level differences or poor viability as neurons expressed FXR2P^EGFP^ at equivalent levels and were healthy for the 4-week observation period with highly elaborate dendritic and axonal arbors (Fig. 2).

We note that the transposon-mediated EGFP insertion resulted in few fusions in the far C-terminal part of the protein (aa456–673). This non-random distribution pattern has been previously shown to occur for this method (Giraldez et al., 2005). Although this distribution could result from a potential sequence insertion bias in transposon behavior (Goryshin et al., 1998), a second possibility is that insertions in these regions of FXR2P were not selected in our initial screening in *E. coli*, which ruled out improperly folded and non-fluorescent FXR2P^EGFP^ fusions.

We currently do not understand the mechanism by which EGFP insertion into discrete regions of the FXR2P LC domain induces fibrillization. However, our knowledge of LC domain structure and function in other proteins suggests several possibilities. Due to their amino acid composition, LC domains are biased towards an unfolded, disordered state. Importantly, upon binding to a molecular partner or through post-translational modifications, these regions can transition to an ordered state where they fold into stable three-dimensional conformations (Dyson and Wright, 2002; Wright and Dyson, 2009, 2015). One possibility then is that the insertion of folded EGFP into the intrinsically disordered FXR2P LC domain triggers such a transition into a highly ordered conformation. In support of this model, FXR2P fragments have been shown to structurally transition and aggregate *in vitro* (Sjekloća et al., 2011). Further, bioinformatic predictions suggest that the FXR2P LC domain contains the intrinsic ability to fibrillize and could undergo a disorder-to-order transition (Fig. 3). While it remains unknown whether our observed fibrillar structures recapitulate endogenous FXR2P conformations, these results indicate that the LC domain is important for regulating FXR2P higher-order structural states. Further, our results support the idea that the LC domain could influence FXR2P to assemble into multiple distinct structural conformations within a neuronal context.

This work identifies four novel FXR2P^EGFP^ assemblages: Type A and Type B fibrils, which each exist both in isolation and in bundles (summarized in Fig. 7O). Ultrastructural analyses show that isolated A and B fibrils have distinct morphologies with unique shapes and diameters (∼20 nm and ∼42 nm, respectively). Type A bundles are thread-like and curvilinear structures that course within the cell body, dendrites and axons at all observed time points (DIV6–28). In contrast, Type B bundles are only found in ≥DIV11 neurons and are straight, needle-like structures that extend radially from cell somata into processes. Interestingly, neither fibril type was observed in the mammalian COS-7 cell line. Importantly, COS-7 cells only expressed the FXR2P^[EGFP]^ constructs for 24 h as compared to weeks in neuron cultures. Given the developmental timeline of both fibril types in neurons, one possible explanation is that fibril formation requires an extended expression period in cells. Alternatively, fibril formation might require additional protein or RNA factors present only in neurons (see below), suggesting a cell-type-dependent expression pattern.

The FXR2P^EGFP^ fibrils described here are morphologically distinct from those observed in previous studies examining other LC domain-containing RBPs. For example, FUS and hnRNPA1/2 form ∼30 nm diameter fibers that are shaped similarly to cross-β structures prototypical of amyloid fibers (Kato et al., 2012; Kim et al., 2013; Schwartz et al., 2013; Hughes et al., 2018; Luo et al., 2018). One possible explanation for the unique morphology of FXR2P^EGFP^ fibrils is a difference in the sequence of its LC domain. The FXR2P LC domain lacks repetitive [G/S]Y[G/S] motifs and QN-rich prion like sequences, both of which are linked to formation of amyloid-like fibrils (Hughes et al., 2018; Luo et al., 2018; Sun et al., 2011; Malinovska et al., 2013). Intriguingly, RBPs that form cross-β fibers are commonly implicated in aggregate pathology observed in neurodegenerative disorders (Ramaswami et al., 2013; Li et al., 2013; Aguzzi and Altmeyer, 2016). However, neurons containing either FXR2P^EGFP^ fibril type survived 28 days in culture without significant cell death.

Our results indicate that LC-dependent structural states can regulate FXR2P^EGFP^ ribosome association. In neurons, ribosomes associate with isolated Type A and B fibrils as well as bundled Type A fibrils. However, ribosomes fail to associate with bundled Type B fibrils (Figs 4–6). Thus, different higher-order FXR2P^EGFP^ structural states show distinct ribosome associations. Notably, endogenous FXR2P-containing assemblages show differential ribosome association in the intact brain. For example, although all FXGs contain FXR2P, ribosomes are only detected in ∼50% of these structures (Akins et al., 2017 and see below). The alternative FXR2P^EGFP^ structural states described here could provide insights into the mechanisms by which the ribosome association of endogenous FXR2P may be regulated in the intact brain.

We speculate that such distinct FXR2P structural states could underlie the circuit-selective formation of FXGs in the intact brain. FXR2P is present in all neuronal cell bodies and proximal dendrites in the intact brain. However, FXGs are only present in select axons, such as corticocortical and thalamocortical fibers, olfactory sensory neurons, hippocampal CA3 associational axons and cerebellar parallel fibers (Christie et al., 2009; Akins et al., 2012). Moreover, four FXG subtypes, all containing FXR2P but differing in FMRP/FXR1P and mRNA content, are present in distinct circuits (Christie et al., 2009; Akins et al., 2017; Chyung et al., 2018). These results suggest that individual neuronal types could contain distinct sets of factors that regulate FXR2P structural organization in a circuit-selective fashion. In support of this hypothesis, we only observed FXR2P fibril formation in neurons and not in the COS-7 cell line, suggesting these regulatory factors could be neuron-specific. Elucidation of the factors promoting FXR2P higher-order structures in neurons could provide important insight into how FXGs and their RNA association are regulated in neuronal subsets in the brain.

Finally, these findings have implications for understanding the role of RNA-binding proteins in neurological disease. Pathological RNA-protein aggregates caused by RBP dysregulation characterize many neurodegenerative diseases including ALS and FTD (Mendoza-Espinosa et al., 2009; Conlon and Manley, 2017; Bowden and Dormann, 2016). Moreover, familial forms of these diseases are often caused by LC domain mutations (Purice and Taylor, 2018). Such mutations are thought to result in dysregulation of endogenous mechanisms controlling higher-order structural states of key RBPs (Kim et al., 2013; Ramaswami et al., 2013; Purice and Taylor, 2018). The regulation of multiple FXR2P higher-order states defined here could thus shed light on structural perturbations mediating abnormal RBP function in neurodegeneration.

## MATERIALS AND METHODS

### Plasmids

Full-length *fxr2* was PCR generated from an *fxr2* clone (Open Biosystems; MMM1013-9498022) and restriction subcloned into the pBluescriptKSII vector. Construction of the modified pCAG plasmid pCAGES and pCAGES-TdTomato has been described previously (Stackpole et al., 2014). The modified Tn5 transposon encoding *EGFP*-*Kan^R^-STOP* (pBNJ24.6) was a kind gift of Dr Thomas Hughes (Sheridan et al., 2002). The FXR2P^[217-ΔLC]^ plasmid was generated by PCR from the FXR2P^[217]^ clone using the forward primer 5′–GAATTCGATGGGCGGCCTGGCC-3′ and reverse primer 3′-CTCGAGTTAAAAGCCCAGCCCAATCTG-5′. The I314N point mutation was generated in FXR2P^[217]^, FXR2P^[416]^ and FXR2P^[435]^ constructs using GeneArt technologies (Thermo Fisher Scientific) with the targeted mutation 5′-GTTAAC-3′ which also introduces an HpaI restriction site for selection during cloning.

### *In vitro* transposition reaction

Transposons were amplified from the pBNJ24.6 plasmid by PCR with a primer complimentary to the Tn5 mosaic end (5′-CTGTCTCTTATACACATCT-3′). The *in vitro* transposition reaction was performed with the amplified transposon and target plasmid (pBluescriptKSII-*fxr2*) using EZ-Tn5 Transposase according to the manufacturer's instructions (Epicentre, Madison, USA). Electrocompetent *E. coli* were transformed with the transposition reaction and plated on LB agar with Kanamycin (30 μg/ml) and Ampicillin (100 μg/ml). To establish transposition efficiency transformation, reactions were plated in parallel on LB agar with Ampicillin alone.

### Generation of full-length EGFP-transposed FXR2 (FXR2P^EGFP^) constructs

To determine which clones harbored the EGFP transposon insert both in-frame and in the correct orientation within pBluescriptKSII-fxr2, colonies were visually screened for EGFP fluorescence using an Olympus SZX12 microscope. A total of 180 fluorescent colonies were selected and DNA was prepared from each clone (miniprep kits from Qiagen, Valencia, USA). Each fluorescent transposed clone was then screened for insertion of EGFP into the *fxr2* coding region (versus vector backbone) by restriction digestion with XbaI and XhoI. Of these, the exact insertion site of the transposon within *fxr2* was identified by sequencing 5′ out of the transposon using a primer complimentary to the EGFP coding region (3′-TTTACGTCGCCGTCCAGCTCGA-5′). To generate full-length fusion proteins, the Kanamycin selection cassette with *STOP* codon was first removed from all clones with unique in-frame insertion sites by digestion with SrfI (Agilent, Santa Clara, USA) and re-ligation with T4 DNA Ligase [New England Biolabs (NEB), Ipswich, USA]. To verify loss of cassette, all colonies were restriction digested with XmaI and KpnI. Each EGFP-transposed *fxr2* construct was then restriction subcloned into the EcoRV and NheI sites of the pCAGES vector to generate pCAGES-FXR2P^EGFP^ constructs.

### Primary rat cortical neuron culture

All animal care and collection of tissue were in accordance with Brown University IACUC guidelines for care and use of laboratory animals. Primary rat cortical neuron cultures were prepared as previously described (Stackpole et al., 2014). For microscopy experiments, cells were plated onto 24-well plates with poly-D-lysine (PDL; 50 μg/ml; Sigma-Aldrich) and laminin (20 μg/ml; Thermo Fisher Scientific) coated glass coverslips (1 mm; Assistant, Germany) at a density of 80,000 cells/well. For western blotting, cells were plated onto PDL-coated 6-well plates at a density of 300,000 cells/well. For electron microscopy, cells were plated onto PDL-coated 4-well Permanox Lab-Tek chamberslides at a density of 80,000 cells/well. Cultures were maintained in an incubator at 37°C and 5% CO_2_ and 95% air.

### Transfection

At DIV3, neuron cultures were co-transfected with pCAGES-FXR2P^EGFP^ constructs along with pCAGES-TdTomato by magnetofection with NeuroMag paramagnetic nanobeads (Oz Biosciences, France). For 24-well plates, plasmid DNA (0.5 μg total, or 0.25 μg each construct) was incubated with 1.75 μl NeuroMag beads for 15 min. For 6-well plates (western blotting), 0.75 µg of plasmid DNA was incubated with 2.62 µl of NeuroMag beads. Solution was then added drop-wise to cultures and allowed to incubate for 15 min on top a magnetic plate within a 37°C incubator. For experiments using COS-7 cells (ATCC, #CRL-1651), cells were split onto PDL-coated glass coverslips and transfected after 24 h using FugeneHD (Promega) according to the manufacturer's instructions.

### Immunostaining

At various time points post transfection, coverslips were washed once with PBS and fixed for 15 min with 4% paraformaldehyde with 4% sucrose in PBS. Coverslips were blocked with PBS with 0.3% Triton X-100 and 1% blocking reagent (Roche, Switzerland) for 30 min and then incubated for 1 h each in the same solution with primary antibody followed, after washing, by secondary antibodies (Thermo Fisher Scientific; 1:1000). Primary antibodies: rRNA 5S and 5.8S subunits were detected using supernatant from hybridoma cells expressing the monoclonal antibody Y10b (gift from Dr J. Twiss, University of South Carolina; 1:200). GFP was detected using antibody N86/8 from NeuroMab (Davis, USA; 1:10). FXR2P was detected with the primary antibody BU38 (1:500; Akins et al., 2012). Coverslips were mounted in NPG (4% n-propyl-gallate, 60% glycerol, 5 mM phosphate pH 7.4, 75 mM sodium chloride). Confocal images were collected on a Zeiss LSM 510 microscope using z-stacks to capture the entire depth of the neuron using a 63× Plan-Apochromat objective. Epifluorescent images of endogenous FXR2P and COS7 cells were collected using Nikon Elements software and a Nikon Eclipse T800 microscope coupled with an Orca ER camera (Hammamatsu, Bridgewater, USA). Images were analyzed using ImageJ and Photoshop CS6 (Adobe). To depict structures that existed across a wide dynamic range, non-linear adjustments were made to brightness and contrast in order to accentuate signal while maintaining background. Quantifications were performed only on images for which linear manipulations were applied uniformly across all images in the dataset regardless of condition.

### *In situ* hybridization

Cultured neurons were washed and fixed as described above and then treated with 0.2 M hydrochloric acid for 10 min and then PBS with 1% Triton X-100 for 2 min. Cultures were rinsed, equilibrated in 2X SSC with 10% formamide, and incubated overnight at 37°C in hybridization solution [10% dextran sulfate, 2 mM vanadyl ribonucleosides (NEB), 2X SSC, 10% deionized formamide, 1 mg/ml *E. coli* tRNA (Roche), 200 µg/ml BSA (Roche)] with oligo(dT)_45_ that had been end-labeled with the DIG oligonucleotide tailing kit following manufacturer's instructions for short tails (Roche). Coverslips were washed with 2× SSC and 10% formamide for 30 min each at 37°C and rinsed with 2× SSC and PBST. A primary antibody against digoxigenin (1:100, Jackson Immunolabs, #200-162-156) was applied to cells in blocking solution for 2 h. Cells were then rinsed with PBST, incubated in secondary antibody for 1 h, washed and mounted in NPG medium and analyzed as described above.

### Bioinformatic predictors of LC domain properties

The FXR2P amino acid sequence was assessed for intrinsically disordered regions using the PONDR-FIT website interface (http://www.disprot.org/pondr-fit.php). For prion-like analyses, the FXR2P amino acid sequence was uploaded to the Prion-Like Amino Acid Composition website (PLAAC; http://plaac.wi.mit.edu/) and analyzed with the default settings. The PLAAC website simultaneously includes analyses using FoldIndex and the 4*PAPA algorithm. For predicting disordered binding regions, the FXR2P amino acid sequence was assessed by ANCHOR using the IUPRED website (http://iupred.enzim.hu/). For above analyses, the graphical output from each website was used for display purposes in this paper. The pi-contact propensity of FXR2P was evaluated using a pi–pi phase separation propensity script (Vernon et al., 2018). The per-residue score of the pi-contact propensity was then plotted in MATLAB. The fibril forming propensity of FXR2P was evaluated using the ZipperDB website interface (https://services.mbi.ucla.edu/zipperdb/) and the Rosetta Energy score was then plotted in MATLAB for display purposes.

### Electron microscopy

DIV6 (FXR2P^[217]^ and ^[416]^) or DIV14 (FXR2P^[435]^) transfected neurons in 4-well chamberslides were washed three times with 1.25% glutaraldehyde in 0.15M sodium cacodylate buffer and fixed overnight at 4°C. A circular diamond scribe objective (Zeiss) was used to score the location of GFP+ transfected neurons (*n*=3 per condition) on the bottom of the chamberslide. Epifluorescent images of each identified neuron were collected with a 10× objective using a Zeiss Axiovert 200M microscope to identify the position of transfected neuron in the scored location. Slides were then rinsed and post-fixed with 1% osmium tetroxide, rinsed and dehydrated through a graded ethanol series. After removal of media chambers and gaskets slides were covered with Epox 812 resin, placed over resin-filled slide-duplicating molds and polymerized overnight. Regions of interest determined from the epiflourescent images were cut out and mounted. Ultrathin sections (50 nm) were collected on wire mesh copper grids incubated with uranyl acetate and lead citrate and examined with a Morgagni 268 transmission electron microscope. Images were collected with an AMT Advantage 542 CCD camera system. The width of isolated fibrils was quantified in ImageJ by measuring the shortest distance across the fibrils perpendicular to the long axis. Depending on the continuous length of the fibrils within individual micrographs, the width was measured and averaged across one to eight sites to give the average diameter of each fibril. Ribosome periodicity was determined in ImageJ by measuring the distance between the centers of adjacent ribosomes along fibrils. Depending on the continuous length of fibrils within individual micrographs, the inter-ribosome distance was measured and averaged across one to six pairs to give the ribosome periodicity for each fibril. Statistics including mean and standard error were calculated in Prism.

### Western blotting

Cultured cortical neurons (DIV3) were transfected as described above. Three days post transfection (DIV6), neurons were washed twice with cold PBS and incubated with RIPA lysis buffer (25 mM Tris pH 8.0, 150 mM sodium chloride, 0.1% SDS, 1% NP-40, 0.5% sodium deoxycholate, 5 mM EDTA, 2 mM sodium orthovanadate, NEB; 10 mM β-glycerophosphate, 1× protease inhibitor cocktail, Roche) for 15 min at 4°C. Lysates were then collected and centrifuged at 10,000 ***g*** for 15 min at 4°C. Supernatants (10 µg) were boiled in SDS-PAGE sample buffer (60 mM Tris pH 6.8, 2% SDS, 10% glycerol, 735 mM β-mercaptoethanol), separated by SDS-PAGE gels and transferred to nitrocellulose membranes following standard methods. Blots were blocked for 1 h at room temperature in 5% non-fat dried milk and 4% normal goat serum in wash buffer (100 mM Tris pH 7.4, 150 mM sodium chloride and 0.1% Tween-20) and then incubated overnight with diluted primaries at 4°C (anti-FXR2P clone 55; 1:1000; BD Biosciences; or anti-γ-actin; 1:40,000, Sigma-Aldrich #A8481). Blots were washed four times for 5 min each in wash buffer after primary incubation. Horseradish peroxidase (HRP)-conjugated secondary antibodies (KPL, Gaithersburg, MD) were diluted in 5% milk in wash buffer (1:2000) and incubated at room temperature for 90 min. Blots were washed four times for 5 min each in wash buffer and signals were detected by chemiluminescence (Amersham ECL western blotting reagents, Arlington Heights, USA).

### Polyribosome analysis

HEK293T cells were cultured in 150-mm culture dishes and transfected with FXR2P^[217]^ or FXR2P^[217::I314N]^ constructs using the calcium chloride method. Briefly, for each 150-mm plate, 45 μg of plasmid DNA was vortexed with 1.2 ml of sterile water and 300 μl of 2.5 M CaCl_2_ followed by drop-wise addition of 1.5 ml of 2X HBS buffer (40 mM Hepes, 274 mM NaCl, 10 mM KCl, 12 mM dextrose, 1.4 mM Na₂HPO₄, pH 7.05). This mixture was vortexed, incubated at room temperature for 20–30 min and then slowly added to the cells. After 4 h, the medium was replaced with fresh medium. Cells were harvested after 24 h by replacing the culture media with fresh media containing cycloheximide (Sigma-Aldrich) at a final concentration of 100 μg/ml for 15 min. Cells were washed twice with ice cold PBS containing 100 μg/ml cycloheximide, trypsinized and pelleted for 5 min at 1000 ***g***. Cells were then resuspended in 750 μl of low-salt buffer (20 mM Tris-HCl pH 7.5, 10 mM NaCl, 3 mM MgCl_2_) followed by incubation on ice for 5 min. Triton-X 100 was added to the cell suspension to a final concentration of 0.3% (v/v) and cells were lysed on ice using a 1-ml Dounce homogenizer. The solution was centrifuged for 1 min at 10,000 ***g*** at 4°C, and supernatants were layered on top of linear 15%–50% (w/v) sucrose gradients, ultracentrifuged (Beckman SW41Ti rotor) at 36,000 rpm for 2 h at 4°C. Polysome profiles were monitored by absorbance of light with a wavelength of 254 nm (*A*_254_). For western blot analysis, 20 μl of each fraction was boiled in SDS-PAGE buffer, separated by SDS-PAGE, transferred to nitrocellulose membrane and then probed by western blot analysis for anti-FXR2P (see above).

## Supplementary Material

Supplementary information
