# Supplementary material for: EGFP insertional mutagenesis reveals multiple FXR2P fibrillar states with differing ribosome association in neurons
Source: Biol Open. 2019 Aug 15;8(8):bio046383. doi: 10.1242/bio.046383 (PMC6737979; doi:10.1242/bio.046383)
Supplement: Supplementary information [file biolopen-8-046383-s1.pdf]

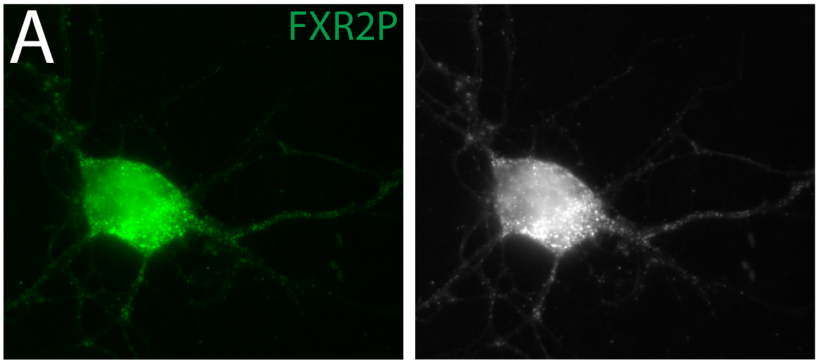

Figure S1. Endogenous FXR2P expression in cultured cortical neurons. *(A)* In the soma of a DIV6 neuron, endogenous FXR2P (green) distributes diffusely in the cytoplasm and nucleus as well as forms granules. In dendrites, endogenous FXR2P localizes to granules.

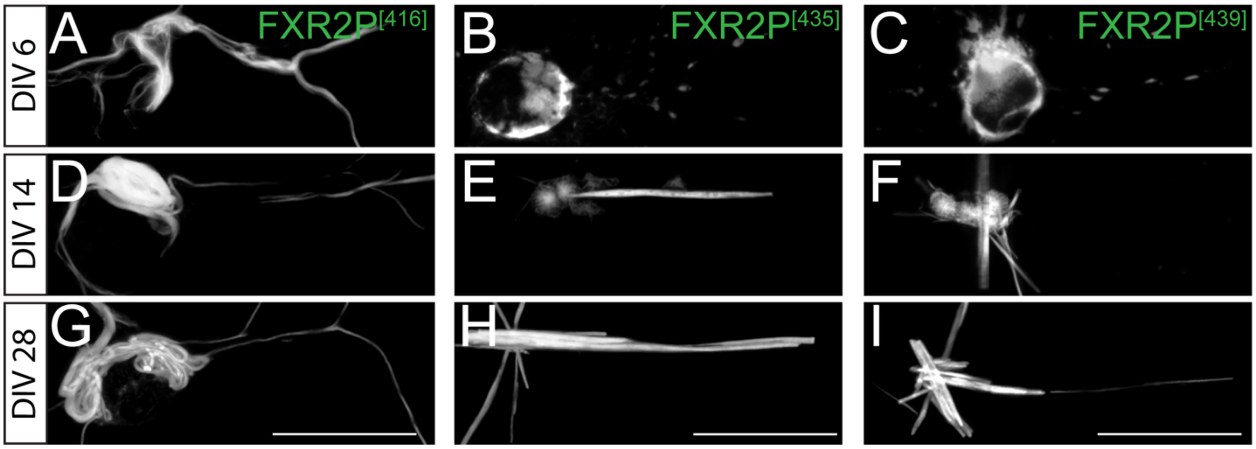

Figure S2. Time course of FXR2P[416], FXR2P[435] and FXR2P[439] in cultured neurons. *(A)* DIV6 neuron expressing FXR2P[416] in Type A fibril bundles both in cell body and dendrite. *(B)* DIV6 neuron showing FXR2P[435] perinuclear localization in soma and within dendritic granules in DIV6 neurons. *(C)* DIV6 neuron expressing FXR2P[439] in a perinuclear distribution as well as within dendritic granules. *(D)* FXR2P[416] is expressed in Type A bundles in DIV14 neurons. *(E)* DIV14 neuron expressing FXR2P[435] in discrete, nestF like structures within cell soma that are closely associated with a Type B fibril bundle. *(F)* FXR2P[439] localizes to spherical, fibrillar structures and Type B fibrils in cell soma of DIV14 neuron. *(G)* In a DIV28 neuron, FXR2P[416] localizes to Type A fibrils in soma that extend into dendrites. *(H)* FXR2P[435] expressed in a DIV28 neuron forms Type B fibrils within soma and dendrites. *(I)* FXR2P[439] forms Type B fibrils in a DIV28 neuron both within soma and dendrites. Scale bar = 20µm.

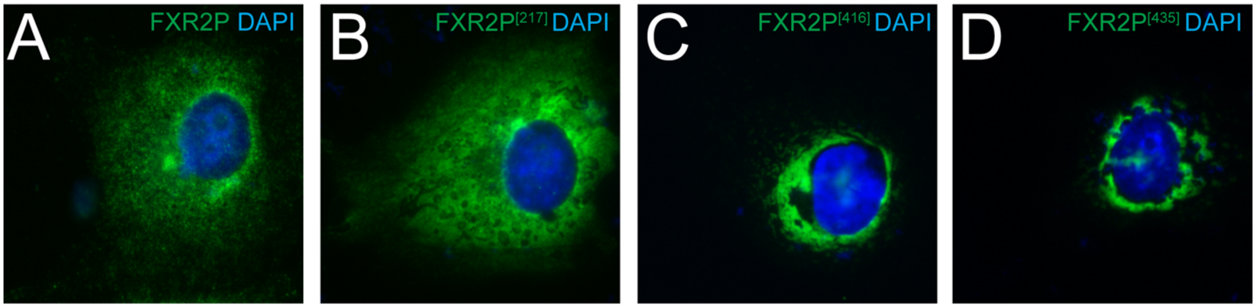

Figure S3. Localization of FXR2P[EGFP] in COSF 7 cells. *(A-B)* Endogenous FXR2P *(A)* and FXR2P[217] *(B)* both distribute diffusely in cytoplasm of COSF 7 cells. *(C-D)* FXR2P[416] and FXR2P[435] show an amorphous, perinuclear distribution in COS-7 cells. No fibrillar structures are observed.
